# Is the association between acne and mental distress influenced by diet? Results from a cross-sectional population study among 3775 late adolescents in Oslo, Norway

**DOI:** 10.1186/1471-2458-9-340

**Published:** 2009-09-16

**Authors:** Jon A Halvorsen, Florence Dalgard, Magne Thoresen, Espen Bjertness, Lars Lien

**Affiliations:** 1 Department of Dermatology, Rikshospitalet University Hospital, Faculty of Medicine, University of Oslo, Norway; 2 Institute of General Practice and Community Medicine, University of Oslo, Norway; 3 Judge Baker Children Center, Harvard Medical School, Boston, USA; 4 Department of Biostatistics, Institute of Basic Medical Sciences, University of Oslo, Norway; 5 Tibet University Medical College, Lhasa, Tibet, PRC; 6 Institute of Psychiatry, University of Oslo, Oslo, Norway

## Abstract

**Background:**

Several studies with conflicting findings have investigated the association between acne and mental health problems. Acne usually starts in adolescents, as does an increase in the prevalence of depression and anxiety. Recently, there has been more focus on the link between diet and acne and diet and mental health problems. The objective of this study is to investigate the association between acne and mental distress and to explore a possible influence of dietary factors on the relation.

**Methods:**

A population-based cross-sectional study in Oslo of 18 or 19 year old adolescents. The participation rate was 80%. Acne was self-reported. To measure mental distress, the Hopkins Symptom Checklist 10 was used. Diet and lifestyle variables were also collected by questionnaire and socio-demographic variables were obtained from Statistics Norway.

**Results:**

The prevalence of acne was 14.4% among the males and 12.8% among the females. The mean score of mental distress increased when the severity of acne increased. In the crude analyses, the significant associations with acne among the males were: mental distress OR = 1.63, frequent consumption of chocolate/sweets OR = 1.40, frequent consumption of potato chips OR = 1.54. The significant crude associations with acne among the females were: mental distress OR = 2.16, infrequent consumption of raw vegetables OR = 1.41, non-Western background OR = 1.77 and low family income OR = 2.14. No crude associations with acne were identified in either gender for the consumption of sugary soft drinks, fatty fish, cigarette smoking or alcohol. In adjusted models which included diet and socio-demographic variables, the association between acne and mental distress was unchanged for both males (OR = 1.68) and females (OR = 2.04), and between acne and infrequent consumption of raw vegetables among the females (OR = 1.38).

**Conclusion:**

Among late adolescents in Oslo, self-reported acne is significantly associated with mental distress and, among girls, with infrequent consumption of raw vegetables. Our finding does not support the hypothesis that dietary factors alter the relationship between acne and mental distress.

## Background

Acne is a very common skin condition of the face and upper trunk affecting millions of adolescents everyday [1]. It is of great interest and importance to explore it further to elucidate possible associated factors which may provide clues to its aetiology.

The distribution of acne in populations has been shown to vary across gender [2-8], ethnicity [1,9], socio-demographic variables [8,10], cigarette smoking [11,12], diet [13,14], mental health problems [15-17] and may even be associated with suicidal thoughts [15,18]. These studies have helped us identify risk factors for the development of acne and made us understand the burden this condition represents for young people. The importance acne may have on the daily life of adolescents must not be trivialized, and it has been demonstrated that the quality of life of acne patients is at the same level as patients with other chronic conditions such as asthma, epilepsy, diabetes, back pain and arthritis [19]

Since acne is a visual disease and starts in adolescents, it is particularly relevant to explore the way in which this skin condition is associated with psycho-social factors. Adolescence is an important and vulnerable period in most people's lives as it is the time of transition between the dependence of childhood to the independence of adulthood [20]. There is, however, conflicting evidence about a coexistence of acne and mental health problems: some studies have found an association [15-17], while others have not been able to identify any [21,22].

Another field of controversy in the study of acne is the way in which lifestyle factors and diet in particular contribute to the distribution of acne in populations. After decades of a lack of studies examining a possible link between diet and acne, there have now been observations that consuming milk can increase the prevalence of acne [13,14] and that a low glycaemic diet can decrease the prevalence of acne [23]. In yet another study of two non-Westernized societies, no cases of active acne among young people was observed: it was discussed whether this finding could be related to lifestyle [24]. This also points to ethnic differences as a field of interest in the study of acne.

Interestingly, in a great majority of cases both acne and mental health problems start in adolescents. Depression and anxiety are common among adolescents, and the increase in prevalence begins at puberty [25]. There is an increasing evidence for a link between mental health problems and diet, e.g. sugar [26], consuming fish [27,28] and other lifestyle factors such as smoking [29]. It has been hypothesized that the same nutritional factors may influence both acne and mental problems [30,31]. In a publication in 2007 by Katzman and Logan they especially mention zinc, folic acid, selenium, chromium and w-3 fatty acids as examples of nutrients that may cause depression, anger and/or anxiety on one side and acne on the other side. In a report of five cases from 2008 both acne and mental health improved after 2-month time period of omega-3-based dietary supplement [31].

In light of this knowledge it is of interest to study acne in a general adolescent population. The aims of this paper are thus first to investigate the association between acne and mental distress and thereafter the possible confounding effect of diet on this relationship.

## Methods

### Participants and study design

The study population consisted of non-healthcare seeking late adolescents in the Norwegian capital of Oslo, a multicultural Western city of about 1/2 million inhabitants. The design of the study was questionnaire-based and cross-sectional. A four-page questionnaire provided information on somatic, mental and lifestyle variables. The study itself (Youth 2004) was conducted by the University of Oslo, the Norwegian Institute of Public Health and the Regional Centre for Child and Adolescent Mental Health, Eastern and Southern Norway. The data were collected at schools and by post [32].

In 2004, all pupils (mainly born in 1986 and thus 18 or 19 years old) in Oslo in their final year of compulsory schooling were invited to participate. This part of the study was conducted in class. Of the 3659 pupils invited, 3308 (90%) participated. In addition, 1085 adolescents in Oslo aged 18 or 19 who were not in their final year were invited by post to participate and 467 (43%) returned the questionnaire. These 1085 adolescents had participated earlier in a population-based study in Oslo [33]. In total, 3775 (80%) of the 4744 adolescents invited participated.

### Measures

#### Acne

This was collected by the question. "In the last week, have you had pimples?" and four possible categories was dichotomized into "No" and "Yes, a little" versus "Yes, quite a lot" and "Yes, very much". The question regarding acne have been assessed in a validation study and also been used in a population study regarding self-esteem and body satisfaction among late adolescents with acne [34,35]. In the validation study the adolescents' answers have been compared to a dermatologist's assessment and the overall agreement, sensitivity and specificity regarding acne was 74%, 93% and 43%.

#### Mental distress

This was measured using Hopkins Symptom Checklist 10 (HSCL-10) which is a ten-item, shortened version of the more widely used HSCL-25, an instrument which mainly measures symptoms of anxiety and depression [36]. The questions in HSCL-10 concern the following symptoms during the last week: "Suddenly scared for no reason, feeling fearful, faintness or dizziness, feeling tense or keyed up, blaming yourself for things, trouble falling asleep, feeling blue, feeling of worthlessness, feeling everything is an effort, feeling hopeless about the future" [37]. Each item is rated on a scale from 1 (not at all) to 4 (extremely). An average score for all the 10 items is calculated and a score equal to or greater than 1.85 has been shown to be a valid predictor of mental health distress in subjects aged 16-24 years old [36]. For inclusion in the total HSCL-10 score, six of the ten questions had to be completed. The missing values were replaced by the sample mean for each value [36].

#### Diet

This was self-reported and sugary soft drinks were dichotomized into "seldom" (never, 1-6 glasses per week, 1 glass daily) versus "often" (2-3 glasses daily, more than 4 glasses daily). The other dietary variables (raw vegetables, fatty fish, chocolate/sweets and potato chips) were all dichotomized into "seldom" (never, 1-3 times a months, 1-3 times a week) versus "often" (4-6 times a week, 1-2 times a day, and 3 or more times a day).

#### Lifestyle variables

These are self-reported variables and cigarette smoking was trichotomized into "never", "has quit" or "sometimes" and "daily". Alcohol was dichotomized into "seldom" (never, sometimes yearly and 1-3 times a month) versus "often" (once a week, 2-7 times a week).

### Socio-demographics

The variable concerning both ethnicity and income was obtained from Statistics Norway which possesses information on all residents in Norway. Non-Western adolescents are defined as having both parents from a non-Western country or as having been born in a non-Western county [38]. Western Europe, North America and Australia are considered to be Western countries in the analyses. Other countries are considered to be non-Western [38]. Income is the total of both parents' gross income and has been divided into three categories: >1.0 million Norwegian kroner (NOK), NOK 1.0-0.5 million, and < NOK 0.5 million. One million NOK is approximately 105,000 Euros.

### Missing

3655 adolescents answered the question about acne, thus 120 missing (3.2%). On the other variables the missing numbers were as follows: mental distress 147, sugary soft drinks 161, raw vegetables 125, fatty fish 137, chocolate/sweets 129 and potato chips 130, cigarette smoking 129, alcohol 174, ethnicity 415, income 708.

### Ethics

The study protocol was evaluated by the Regional Committee for Medical Research Ethics and approved by the Norwegian Data Inspectorate and the educational authorities in Oslo. It has been conducted in full accordance with the World Medical Association's Declaration of Helsinki. Written, informed consent was obtained from all the participants.

### Statistics

SPSS for Windows version 14.0 was used for the statistical analyses. Odds ratios (OR) were calculated in both crude and adjusted logistic regression models. The level of significance was set at p < 0.05 and 95% CI were calculated.

## Results

The population characteristics in our study showed a slight preponderance of females (55.7%) and the majority of the adolescents come, as expected, from middle income families (53.1%) and have a Western background (76.4%). (Table 1).

**Table 1:** Population characteristics of the sample of adolescents (N = 3775)

		**N**	**%**
Gender			
	Boys	1672	44.3%
	Girls	2103	55.7%
			
Income			
	> 1.0 million NOK*	729	23.5%
	0.5-1.0 million NOK	1651	53.1%
	< 0.5 million NOK	728	23.4%
			
Ethnicity			
	Non-Western	806	23.6%
	Western	2611	76.4%

* 1.0 million NOK is approximately 105,000 Euros

**Figure 1:**
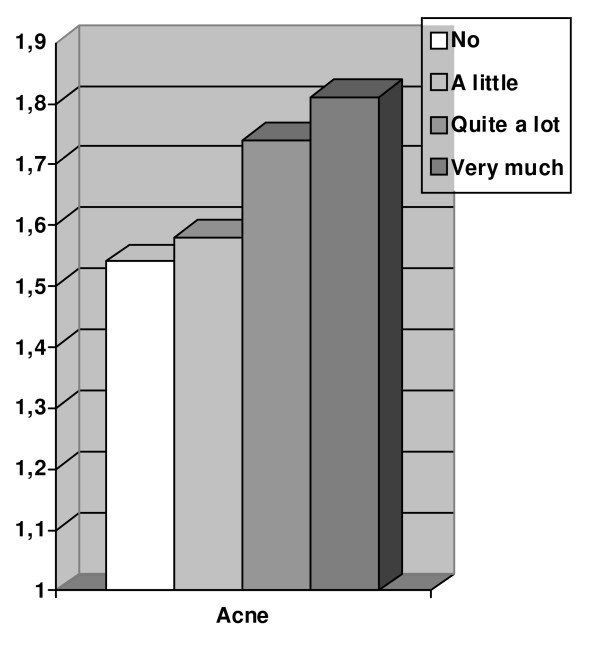
**Mean scores on HSCL-10 (The 10 item Hopkins Symptoms Check List) with increasing severity of self-reported acne (no, a little, quite a lot and very much) in late adolescence among both genders in Oslo, Norway.**

The prevalence of acne in the sample was 13.5% (95%CI; 10.5-16.5), reported non-significantly more often among the males than the females. The prevalence of acne among males with mental distress was 20.5% (95%CI; 15.3-25.7%) which is significantly higher than among males without mental distress, 13.4% (95%CI; 11.6-15.2%). The prevalence of acne among females with mental distress was 18.8% (95%CI: 15.9-21.1%) which is significantly higher than among females without mental distress, 9.6% (95%CI: 8.0-11.2%) (Table 2). These associations are also reflected in the estimated odds ratios in table 3. In an adjusted model including diet, lifestyle and socio-demographic factors, the adjusted association between acne and mental distress among the males showed an OR = 1.68 (95%CI:1.10-2.57) and among the females an OR = 2.04 (95%CI:1.50-2.77). When calculating the mean score of HSCL-10 in the four categories of acne, the mean score increased for each category when acne became more severe (Figure 1).

**Table 2:** Prevalence of self-reported acne (quite a lot and very much) across mental distress, diet, lifestyle and socio-demographic factors among 1672 boys and 2103 girls in a cross-sectional study in Oslo, Norway (n = 3775).

		**Acne**			
		**Males**		**Females**	
		**N**		**N**	
Whole sample		1612	14.4% (12.7-16.1)	2043	12.8% (11.4-14.3)
Mental distress					
	No	1371	13.4% (11.6-15.2)	1327	9,6% (8.0-11.2)
	Yes	229	20.5% (15.3-25.7)	701	18.8% (15.9-21.1)
					
Diet					
Soft drinks with sugar	Often	405	13.8% (10.4-17.2)	177	11.9% (7.1-16.7)
	Seldom	1196	14.7% (12.7-16.7)	1836	13.0% (11.5-14.5)
Raw vegetables	Often	597	12.7% (10.0-15.4)	999	10.8% (8.9-12.7)
	Seldom	1011	15.2% (13.0-17.4)	1043	14.7% (12.5-16.9)
Fat fish	Seldom	1482	14.1% (12.3-15.9)	1918	12.4% (10.9-13.9)
	Often	123	16.3% (9.8-22.8)	115	17.4% (10.5-24.3)
Chocolate/sweets	Seldom	1124	13.0 (11.0-15.0)	1379	11.7 (10.0-13.4)
	Often	484	17.3 (13.9-20.7)	659	15.2 (12.5-17.9)
Potato chips	Seldom	1361	13.6 (11.8-15.4)	1804	12.5 (11.0-14.0)
	Often	246	18.3 (13.5-23.1)	234	14.5% (10.0-19.0)
					
Lifestyle					
Cigarette smoking	Yes, daily	281	13.2% (9.2-17.2)	418	10.5% (7.5-13.5)
	Yes, but has quit or sometimes	450	14.4% (11.2-17.6)	577	13.0% (10.3-15.7)
	No, never	878	14.8% (12.4-17.2)	1042	13.5% (11.4-15.6)
Alcohol intake	Often	715	12.7% (10.3-15.1)	710	10.8% (8.5-13.1)
	Seldom	882	15.6% (13.2-18.0)	1294	13.7% (11.8-15.6)
					
Socio-demographic factors					
Ethnicity	Western	1143	14.5% (12.5-16.5)	1441	10.8% (9.2-12.4)
	Non-Western	322	14.0% (10.2-17.8)	454	18.5% (14.9-22.1)
Income	>1.0 million NOK	335	13.1% (9.5-16.7)	388	8.2% (5.5-10.9)
	0.5-1.0 million NOK	729	14.5% (11.9-17.1)	905	12.2% (10.1-14.3)
	< 0.5 million NOK	282	14.9% (10.7-19.1)	428	17.1% (13.5-20.7)

95% confidence interval

**Table 3:** Odds ratio for reporting acne with mental distress, diet, lifestyle factors and socio-demographic variables among 1316 and 1647 late adolescent boys and girls in Oslo, Norway.

		**Acne**			
		**Crude OR**		**Adjusted OR**	
		**Males**	**Females**	**Males**	**Females**
					
Mental distress					
	No	1.0	1.0	1.0	1.0
	Yes	1.63 (1.08-2.44)	2.16 (1.61-2.90)	1.68 (1.10-2.57)	2.04 (1.50-2.77)
					
Diet					
Soft drinks with sugar	Often	1.0	1.0	1.0	1.0
	Seldom	1.22 (0.84-1.77)	0.88 (0.53-1.46)	1.04 (0.95-2.08)	1.11 (0.64-1.92)
Raw vegetables	Often	1.0	1.0	1.0	1.0
	Seldom	1.26 (0.91-1.74)	1.41 (1.05-1.90)	1.30 (0.93-1.82)	1.38 (1.01-1.88)
Fat fish	Seldom	1.0	1.0	1.0	1.0
	Often	1.49 (0.81-2.67)	1.47 (0.81-2.67)	1.57 (0.86-2.86)	1.32 (0.70-2.47)
Chocolate/sweets	Seldom	1.0	1.0	1.0	1.0
	Often	1.40 (1.01-1.94)	1.29 (0.95-1.74)	1.26 (0.84-1.88)	1.26 (0.84-1.88)
Potato chips	Seldom	1.0	1.0	1.0	1.0
	Often	1.54 (1.03-2.29)	1.07 (0.67-1.70)	1.45 (0.88-2.40)	1.45 (0.88-2.40)
					
Lifestyle					
Cigarette smoking	Yes, daily	1.0	1.0	1.0	1.0
	Yes, but has quit or sometimes	1.06 (0.65-1.74)	1.12 (0.73-1.73)	1.16 (0.70-1.92)	1.25 (0.80-1.95)
	No, never	1.14 (0.73-1.78)	1.09 (0.73-1.61)	1.19 (0.73-1.93)	1.18 (0.77-1.82)
Alcohol intake	Often	1.0	1.0	1.0	1.0
	Seldom	1.31 (0.95-1.79)	1.22 (0.90-1.66)	1.33 (0.93-1.89)	1.01 (0.71-1.43)
					
Socio-demographic factors					
Ethnicity	Western	1.0	1.0	1.0	1.0
	Non-Western	1.02 (0.69-1.51)	1.77 (1.27-2.45)	0.79 (0.48-1.31)	1.53 (0.95-2.16)
Income	>1.0 million NOK	1.0	1.0	1.0	1.0
	0.5-1.0 million NOK	1.09 (0.68-1.73)	1.55 (1.02-2.34)	1.0 (0.57-1.74)	1.33 (0.87-2.04)
	< 0.5 million NOK	1.10 (0.76-1.61)	2.14 (1.36-3.35)	1.06 (0.72-1.57)	1.48 (0.88-2.48)

95% confidence interval

Adjusted OR for reporting acne with mental distress, adjusted for all other factors in the table among 1316 and 1647 late adolescent boys and girls in Oslo Norway.

Five different dietary variables were included in the analyses as possible confounders. In the crude analyses among the males, acne was associated with the consumption of chocolate/sweets with an OR = 1.40 (95%CI: 1.01-1.94) and the consumption of potato chips with an OR = 1.54 (95%CI: 1.03-2.29). However, these associations disappeared in the adjusted model. In the crude analyses among the females, low consumption of raw vegetables was associated with acne with an OR = 1.41 (95%CI: 1.05-1.90) and this association remained significant also in the adjusted model with OR = 1.38 (95% CI: 1.01-1.88). (Table 3). No other significant associations between acne and diet were identified, but there was a tendency that acne was more prevalent among those with a high intake of fatty fish.

Regarding lifestyle factors, the prevalence of acne was a little lower among those who smoke cigarettes and among the adolescents who frequently consume alcohol. This was seen in both genders, but these findings are non-significant (Table 2).

Socio-demographic factors were associated with acne among the females in the crude model, but not among the males. Among the females, a non-Western background was associated with acne with an OR = 1.77 (95%CI 1.27-2.45), but this association did not remain significant in the adjusted model. Females from low-income families had more acne as seen in Table 2 and the association in the crude model showed an OR = 2.14 (95%CI: 1.36-3.35).

Other models with different combinations of the variables used in the adjusted analyses were tried out, but none of these altered the estimated association between acne and mental distress in any direction. No collinearity was identified between the variables. We also tested for interaction, and no statistical interaction was identified between gender and mental distress or between ethnicity and mental distress.

## Discussion

In this population-based study of a general adolescent population, the strongest and most consistent finding is the significant association between acne and mental distress which is found among both boys and girls in the crude and the adjusted model. Another result from our study that supports a link between acne and mental distress is the increase in mental distress when the severity of acne increases. These two findings have to our knowledge never been seen before in the same study.

Previous studies have also shown an association between mental problems and acne. In a large study in New Zealand of 9567 adolescents of 12-18 years of age, a significant odds ratio of 2.04 was seen between self-reported acne and depressive symptoms measured using Reynolds Adolescent Depression Scale [15]. In Australia, Kilkenny et al found an association (OR = 1.61) between self-reported severe acne and psychiatric symptoms measured using the Clinical Interview Schedule among 2491 students [17]. From the United Kingdom, Smithard et al found among 317 pupils aged 14-16 a significant odds ratio of 1.86 between acne which was objectively assessed by a researcher and psychological morbidity, measured by Strength and Difficulties Questionnaire [16].

These studies are in contrast to a recent Finnish study of 165 male conscripts with acne who did not suffer more depressive symptoms than patients with knee symptoms [22]. The Leeds acne grading scale was used to objectively measure acne, and mental problems were measured with the General Health Questionnaire and the Beck Depression Inventory. In yet another study of 2657 students aged 14 to 20 from Turkey, no association between acne and mental health was identified [21]. Acne was objectively assessed by the Global Acne Grading System and mental health by the Hospital Anxiety and Depression scale in the Turkish study.

The reason for these conflicting results is not obvious, but since there are many different instruments used to carry out the measurements and low number of participants in some of the studies, it is hard to compare the results. However, there are other observations that point to a link between mental health problems and acne. First of all, acne is a visual condition and may therefore cause a variety of psychosocial effects such as decreased self-confidence, social impairment, depression and anger [39]. Second, it is possible that mental health problems cause or increase acne. This is probably not as obvious, but there are several points that may support this view. Stressful events can exacerbate acne, as shown in a sample of 22 university students [40]. The prevalence of mental distress is very high in late adolescence when acne is very common. From Oslo the prevalence of mental distress among 15-16 year old adolescents are 18%, in the present study (18-19 year old adolescents) 26% and among adults 13% [41,42]. It is suggested that neurogenic factors could contribute to the onset or exacerbation of acne formation, possibly via the neuropeptid substance P and increased number of nerve fibres around the sebaceous glands in acne patients [43]. Stress can elicit substance P from peripheral nerves and thereafter may accelerate lipogenesis in the sebaceous glands [43]. The increase in nerve fibres in acne-prone skin may result from raised expression of nerve growth factor on the sebaceous glands which act as a neurotrophic molecule stimulating the sprouting of nerve fibres in the skin [43]. In addition receptors for corticotrophin-releasing hormones have been identified on human sebocytes, especially acne-involved skin [44,45]. Corticotrophin-releasing hormone can be one neuroendocrine factor that contributes the development and exacerbation of acne [44]. It has also been reported that antidepressive medication can improve acne [46]. Finally, there may be one or more common factors influencing both acne and mental problems. Medication can be one such factor, and the widely used drug for acne, isotretionoin, has been linked to an increase in depression, but no causal relationship has been established [47,48]. Unfortunately neither in the present study nor in other studies exploring the association between acne and mental problems [15-17,21,22] is isotretinoin introduced as a possible confounding factor. However, information from the Norwegian Prescription Database shows that 27 individuals living in Oslo, 5 girls and 22 boys, born in 1986 were dispensed isotretinoin at a pharmacy in 2004. It is not very likely that such a low number of users could explain the main finding in this study. Finally, another variable that could influence both acne and mental health is diet [30].

In the present study an association between acne and a low intake of raw vegetables and a high intake of chocolate/sweets and potato chips has been found, but in the adjusted model the association with raw vegetables remains significant only among girls. This has never been shown before. Recently there has been renewed interest in the link between acne and diet. A small randomized controlled study has shown that a low glycaemic diet decreases the number of acne lesions after 12 weeks [23]. Interestingly the protective effect of a high intake of vegetables and low intake of chocolate/sweets and potato chips in the present study is in coherence with the hypothesis that a low-glycemic diet is beneficial to arrest the development of acne. However, we did not find that high intake of fish had a protective role in the development of acne, contrary to what has been suggested [30,31]. Longitudinal observations have discovered that a high intake of milk is associated with acne [13,14]. Unfortunately we did not have data on the intake of milk in this study, and theoretically milk could have been a confounder in the exploration of the association between acne and mental distress. However, milk is probably not a confounder, since there is no established evidence for an association between consuming milk and mental problems. We have not found any studies that compare acne prevalence across populations with different diet. Our findings do not support the idea that diet influences the association between acne and mental health problems.

In the study we have found an almost doubled prevalence of self-reported acne among girls with a non-Western background compared to the reference population. Also among girls, acne is more frequently reported in those who have a low income. It has also been shown previously that acne can be associated with socio-demographic variables [8,9,17,49]. In the adjusted analyses, the association between the socio-demographic variables and acne is no longer significant. This highlights the importance of including relevant confounders, e.g. mental problems, in such models.

Cigarette smoking and the intake of alcohol were in our study either associated with acne. Regarding smoking our results are in correspondence with a study from Denmark among girls [50], but in contrast to studies showing both increased [11] and decreased prevalence of acne in smokers [12]. No studies in the literature were found concerning acne and alcohol intake.

The major limitation of this study is the cross-sectional design which limits the interpretation of the direction of the associations. Further, collecting data using a questionnaire may be problematic, especially problematic concerning diet, but there is also the possibility of dependent misclassification [51]. The strength of the study is the non-healthcare-seeking sample with a relatively high rate of participation (80%), the use of validated instruments to measure acne and mental distress [35,36] and the inclusion of possible confounders in the multivariate analyses which are lacking in similar studies [15-17,21,22].

## Conclusion

In this study a consistent association between acne and mental problems has been shown for both genders in a model which also includes diet, lifestyle factors and socio-demographic variables. The findings do not support the hypothesis that diet may have a confounding effect on the association between acne and mental problems. This study points to mental problems as an important factor in acne, but the causal relationship remains elusive. Therefore we would suggest that measuring psychological parameters should be included in future studies on acne, especially in studies with a longitudinal design. Acne has been shown to have high heritability in twin studies [52]. However, it is important to be aware that this does not automatically imply that environmental factors have no influence. If the whole population is exposed to the same risk factors, genetic factors will emerge as a strong determinant of disease. The fact that acne is rarely seen in some non-Western societies [24] should encourage a further search for risk factors. This study shows that both dietary factors and mental health problems are candidates as causative factors in the development of acne among adolescents in a Western country.

## Competing interests

The authors declare that they have no competing interests.

## Authors' contributions

JAH carried out the statistical analyses and drafted the manuscript. FD took part in planning the study, designed part of the questionnaire and commented on the drafts. MT took part in the statistical analyses and commented on the draft. EB took part in planning the study, participated in its design and coordination, and commented on the draft. LL commented on the statistical analyses and the draft. All authors read and approved the final manuscript.

## Pre-publication history

The pre-publication history for this paper can be accessed here:


